# Comparative Analysis of Sauced Beef Based on Electronic Nose, GC-IMS, and Fuzzy Comprehensive Evaluation

**DOI:** 10.3390/foods15213961

**Published:** 2026-11-04

**Authors:** Zhenxia Cao, Bing Yang, Wanli Zhang, Jing Yan, Jingwen Jia, Minnan Liu, Shuanshuan Xue, Jiaqi Sun, Yingjian Hou, Yating Dai, Heng Wang, Lishui Chen

**Affiliations:** Food Laboratory of Zhong Yuan, Luohe 462300, China; caozhenxia@zyfoodlab.cn (Z.C.);

**Keywords:** sauced beef, GC-IMS, E-nose, VOCs, flavor characterization

## Abstract

Sauced beef is a traditional Chinese meat product, yet the volatile and sensory signatures of potential aroma-active compounds remain insufficiently characterized. Five commercial sauced beef products (B1–B5) were analyzed by electronic nose (E-nose), gas chromatography–ion mobility spectrometry (GC-IMS), chemometrics, and fuzzy comprehensive evaluation (FCE). Volatile organic compounds (VOCs) identified by GC-IMS were screened by principal component analysis (PCA) and partial least squares discriminant analysis (PLS-DA). An FCE model based on texture profile analysis (TPA) was established, with color, taste, flavor, texture, and acceptability as sensory evaluation indicators. E-nose responses indicated that sulfur-containing compounds, aromatic components, alkanes, and alcohols contributed strongly to overall aroma. GC-IMS tentatively identified 73 VOCs, with ketones, alcohols, and aldehydes as the dominant classes. Dual screening (variable importance in projection (VIP) > 1 and odor activity value (*OAV*) ≥ 1) identified 14 potential aroma-active compounds. Nine compounds were common to all five products, and five were detected in only a subset of products. Under equal weighting, the fuzzy evaluation ranked overall quality as B2 > B4 > B3 = B5 > B1. Pearson correlation revealed that sensory attributes, especially taste and flavor, were positively correlated with 3-(Methylsulfanyl)propanal and (Z)-2-pentenal, while negatively correlated with (R/S)-linalool, 2-Butanone-3-hydroxy, and alpha-Pinene. These integrated approaches revealed differences among the five commercial products investigated, identified potential aroma-active compounds, and support product-specific characteristics and quality assessment of sauced beef products.

## 1. Introduction

With a centuries-long history, sauced beef holds a prominent position among traditional Chinese meat products. The production process involves cooking beef in a broth enriched with various spices and seasonings, followed by extended simmering to impart characteristic sensory properties and improve product shelf life [1]. The flavor of sauced beef arises from a complex interplay of endogenous precursors in the raw meat and exogenous contributions from spices and seasonings, mediated by thermal reactions such as the Maillard reaction, Strecker degradation, and lipid oxidation [2]. Because each manufacturer selects different beef breeds and cuts, employs proprietary spice blends, and adopts distinct curing, stewing, and sterilization protocols, commercial sauced beef products can differ markedly in aroma, taste, and texture even within the same product category [3]. These differences directly affect consumer acceptance and market competitiveness, yet the specific volatile and sensory signatures that distinguish one brand from another remain poorly characterized. With the continuous optimization of meat processing technologies, consumer expectations have shifted from concerns about variety and quantity to demands for quality. Consequently, how to effectively analyze and evaluate the quality of sauced beef has become a key issue for both producers and consumers.

Flavor is one of the most important indicators for evaluating the quality and overall acceptability of meat products. It primarily originates from non-volatile taste-active components and volatile aroma compounds. Exogenous spices (e.g., star anise, fennel, clove) and seasonings (e.g., soy sauce, soybean paste) introduce additional VOCs and participate in flavor-generating reactions during prolonged stewing, further complicating the endogenous chemical reaction network [3]. The sensory impact of VOCs depends on both their concentration and olfactory threshold; the *OAV* effectively captures this relationship [4]. Even trace amounts of potent odorants may dominate the overall aroma perception. Most studies on the flavor quality of sauced beef have focused on the effects of different detection methods, beef cuts, cooking methods, and processing technologies [5,6]. China, as a leading beef producer, has numerous regional sauced beef styles and brands, each with its own characteristic aroma profile. However, systematic cross-brand comparisons of commercially available sauced beef products remain scarce.

GC-IMS is a green, efficient, and sensitive analytical technique that enables rapid separation and identification of volatile organic substances in complex mixtures [7]. GC-IMS has been widely applied in food analysis and has proven particularly valuable for monitoring and characterizing VOCs in meat products [8,9]. As an auxiliary tool equipped with biomimetic sensor arrays, E-nose replicates human olfactory perception, enabling rapid characterization of food aroma profiles [10]. Numerous studies have combined E-nose and GC-IMS with chemometrics for aroma evaluation of food products [11,12]. The integration of these techniques enables stepwise screening of key aroma markers with distinct discriminatory ability from a broad spectrum of VOCs, thereby facilitating a more comprehensive and reliable analysis of food aroma.

Food sensory analysis relies on human senses to evaluate samples. Traditional sensory evaluation methods, however, often fail to precisely capture changes in food quality, and scoring criteria may not accurately reflect the membership degree of food to sensory grades [13]. Various subjective factors affect sensory evaluation, among which the fuzziness of sensory assessment is the most prominent [14]. The FCE method combines fuzzy mathematics with comprehensive evaluation, handling complex problems with multiple factors and indicators. Applying fuzzy mathematics to food sensory evaluation renders the results more scientific and objective [15], as it better accommodates the uncertainty of panelists’ subjective judgments and avoids the influence of subjective factors inherent in traditional scoring methods [16]. Previous studies on sauced beef flavor have mostly focused on the effects of processing conditions [17] or regional flavor differences [9,18,19]. Although E-nose, GC-IMS, and multivariate statistical analysis have been widely applied to characterize the volatile profiles of sauced meat products, the combined characterization of volatile composition, textural properties, and comprehensive sensory attributes remains worthy of further investigation. Therefore, this study integrated instrumental volatile profiling, TPA, and FCE to investigate five commercial sauced beef products. By incorporating objective textural measurements and comprehensive sensory assessments alongside volatile profiling, this study provides a more multidimensional description of product-specific quality characteristics and preliminary insights into product differentiation.

## 2. Materials and Methods

### 2.1. Materials

Based on online and offline market surveys, five brands of sauced beef with high consumer ratings and large sales volumes were selected as the research subjects. All these sauced beef products were vacuum pre-packaged and purchased from Taobao Mall. The samples from different brands were designated as B1–B5, with detailed information presented in Table 1. For each brand, products manufactured within the same production batch were selected. The production dates of all tested samples varied by no more than 15 days. Samples with similar production dates were randomly selected, and prior to experimental analysis, all samples were stored at room temperature in accordance with the manufacturer’s storage requirements.

### 2.2. Main Reagents

n-ketone standards: 2-Butanone, 2-Pentanone, 2-Hexanone, 2-Heptanone, 2-Octanone, and 2-Nonanone (all of analytical grade), purchased from Shanghai Aladdin Biochemical Technology Co., Ltd. (Shanghai, China); nitrogen gas (purity ≥ 99.999%), obtained from Shanghai Yuanye Bio-Technology Co., Ltd. (Shanghai, China); and 2-Octanol (analytical grade), also obtained from Shanghai Yuanye Bio-Technology Co., Ltd.

### 2.3. Instruments and Equipment

Electronic nose PEN3 (AirSense Analytics GmbH, Schwerin, Germany); 20 mL headspace vials (Shandong Hanon Scientific Instrument Co., Ltd., Dezhou, Shandong, China); MXT-WAX capillary column (15 m × 0.53 mm, 1.0 μm; Restek Corporation, Bellefonte, PA, USA); FlavourSpec^®^ gas chromatography–ion mobility spectrometer (G.A.S. GmbH, Dortmund, Germany); texture analyzer (AMETEK Brookfield, Middleboro, MA, USA); JA2203X analytical balance (Tianjin De’ante Sensing Technology Co., Ltd., Tianjin, China); food pulverizer (Cixi Naiou Electric Appliance Co., Ltd., Cixi, Zhejiang, China); digital constant-temperature water bath WB-1 (Changde Bikeman Biotechnology Co., Ltd., Changde, Hunan, China); intelligent high-performance centrifuge Avanti JXN-24L004 (Beckman Coulter, Inc., Brea, CA, USA).

### 2.4. Sample Preparation

After removing the packaging, the beef samples were stripped of surface jellies and fasciae, then ground into granules using a laboratory meat grinder (Cixi Naiou Electric Appliance Co., Ltd., Cixi, Zhejiang, China). The prepared samples were sealed and stored under refrigeration at 4 °C (not frozen); the maximum storage time before analysis was 12 h; after opening, samples were ground and analyzed within 2 h (E-nose) and 4 h (GC-IMS) to minimize volatile loss.

### 2.5. E-Nose Analysis

The E-nose measurements were conducted using a slightly modified version of the procedure reported by Sun J et al. [1]. Approximately 4.00 ± 0.01 g of minced beef was placed into a 50 mL beaker and sealed with sealing film. The sample was allowed to equilibrate at room temperature for 30 min, then immersed in a water bath at 45 °C for 2 min prior to measurement. The operating conditions were set as follows: a headspace sample detection time of 90 s, a sensor cleaning time of 120 s, a flow rate of both the headspace sample and clean air at 0.6 L/min, and a headspace sample injection vacuum of 10 mL. Each beef sample was analyzed in triplicate, and these replicates were obtained from three independent packages originating from the identical sample batch. All operations were performed at room temperature (25 ± 1 °C). The portable PEN3 E-nose (AirSense Analytics GmbH, Schwerin, Germany) is equipped with 10 different sensors (AirSense Analytics GmbH, Schwerin, Germany) (detailed in Table 2).

### 2.6. HS-GC-IMS Analysis

GC-IMS analysis followed the procedure reported previously, with minor alterations [22]. Headspace autosampling conditions: Approximately 2.0 g of each sample was transferred into a 20 mL headspace vial, followed by the addition of 20 μL of 2-Octanol (100 ppm) as the internal standard. Each sample was analyzed in triplicate. The three replicates were obtained from three independent packages originating from the identical sample batch. Samples were incubated at 60 °C for 15 min under agitation at 500 r/min. A 500 µL volume of headspace gas was introduced into the instrument without splitting, while the injection needle was held at 105 °C. For semi-quantification, the concentration of VOC i in the sample was estimated as C_i_ = (A_i_/A_IS_) × (C_IS_ × V_IS_)/(RF_i_ × m_s_), where C_i_ is the estimated concentration (μg/kg); A_i_ and A_IS_ are the peak areas of VOC i and 2-Octanol, respectively, recorded in the same GC-IMS signal units; C_IS_ is the concentration of the internal-standard solution (μg/μL); V_IS_ is the added internal-standard volume (μL); and m_s_ is the sample mass (kg). RF_i_ is the unitless relative response factor defined as (A_i_/A_IS_)/(m_i_/m_IS_), where m_i_ and m_IS_ are the masses of VOC i and 2-Octanol, respectively, expressed in the same mass units (e.g., μg). Because analyte-specific calibration curves and response factors were not available, RF_i_ was assumed to equal 1 for all VOCs. If 100 ppm denotes 100 μg/mL, the 20 μL addition corresponds nominally to 2.0 μg of 2-Octanol. The resulting values are semi-quantitative; the unit-response-factor assumption introduces compound-dependent uncertainty, so comparisons among products for the same VOC are exploratory and values should not be interpreted as absolute comparisons among different VOCs.

GC conditions: The analytical column was maintained at 60 °C throughout the separation. Nitrogen with a purity of at least 99.999% served as the mobile phase. The flow program was as follows: the carrier-gas flow was initially maintained at 2.0 mL/min for 2 min, followed by a gradual increase to 10.0 mL/min over the next 8 min. The flow was then raised to 100.0 mL/min during the subsequent 10 min and kept constant for another 20 min, resulting in a total analytical duration of 40 min. The injection port temperature was 80 °C.

IMS conditions: A tritium (^3^H) source was used as the ionization source. The drift tube length was 98 mm, and operated under an electric field of 500 V/cm at 45 °C. Ultra-high-purity nitrogen (≥99.999%) was supplied as the drift gas at 150.0 mL/min. Measurements were acquired with the instrument configured for positive-ion detection.

Identification and analysis of VOCs: A standard mixture containing six n-ketones (C4-C9) was analyzed under the specified GC-IMS conditions to determine retention indices (RI) based on chromatographic retention behavior. Identification of the VOCs was assigned by searching and matching against the GC retention index database (NIST 2020) and the IMS drift time database incorporated into the VOCal software (version 0.4.03, G.A.S., Germany).

### 2.7. OAV Analysis

*OAV* has been widely used to estimate the odor contribution of aroma compounds. Typically, compounds with an *OAV* ≥ 1.0 are considered major contributors to the overall flavor [4]. The *OAV* of each volatile compound was determined by dividing its mass concentration by the corresponding odor threshold, as expressed in the following equation:

$$\mathrm{OAV}=\frac{C}{T}$$

where *C* is the determined content of the volatile compound (μg/kg), and *T* is the odor threshold of the same compound in an air medium (μg/kg).

Although air-phase odor thresholds were selected based on the headspace sampling characteristics of HS-GC-IMS, the sample-mass-normalized semi-quantitative VOC concentrations were not directly equivalent to gas-phase concentrations. Therefore, the calculated *OAVs* were used only as exploratory indicators of potential aroma relevance, and their absolute values should be interpreted with caution.

### 2.8. Textural Properties Analysis

Beef samples were cut along the muscle fiber direction into cubes of 10 × 10 × 10 mm^3^, and analyzed using a texture analyzer equipped with a P/36 probe. The following parameters were applied: pretest and post-test speed 1.0 mm/s, a trigger force of 5 g, compression ratio of 30%, return height 20 mm, and time interval between measurements 5 s. Three independently packaged beef samples from the same production batch were used for TPA. A total of eight uniformly sized cubes were randomly selected (three, three, and two cubes per package, respectively). The measurements were averaged within each package, and the package-level means were used as independent replicates (*n* = 3) for statistical analysis. The measured parameters included hardness, springiness, cohesiveness, gumminess, and chewiness.

### 2.9. Fuzzy Comprehensive Sensory Evaluation Method for Sauced Beef

#### 2.9.1. Sensory Evaluation

The FCE method is an effective multi-factor decision-making approach for the comprehensive assessment of objects influenced by multiple factors. This method can significantly reduce the impact of subjective human judgment, thereby yielding more scientific evaluation results. A sensory panel comprising ten assessors (five females and five males), aged 26–35 years, was assembled, with each participant having more than one year of experience in sensory evaluation. Each panelist was required to refrain from consuming any foods that might affect olfaction and gustation for 12 h prior to the sensory evaluation session. The evaluation was conducted at room temperature. The five samples were cut into 10 × 10 × 10 mm^3^ cubes and placed on white plates labeled with three random digit codes, and presented to the assessors in randomized order to minimize potential order and identification biases. The panelists performed sensory evaluations of the beef products from each group, using warm water to cleanse their mouths between successive samples. Each sample was evaluated in triplicate across separate sessions to ensure independence of evaluations. Using the FCE method, the scoring criteria were based on the “General Quality for Soy Sauce and Pot-Roast Meat Products” (GB/T 23586-2022) [20] with slight modifications according to previously published methods [1]. Five attributes were assessed: color, taste, flavor, texture, and acceptability, each with three grades. The detailed sensory scoring criteria are presented in Table 3. The aim was to obtain comprehensive evaluation scores and rankings for each group of beef samples, thereby objectively assessing the product quality of different brands of sauced beef. The sensory evaluation was intended to describe product characteristics rather than consumer preferences or acceptance.

#### 2.9.2. Establishment of Sensory Evaluation Factors and Comment Set

Five sensory attributes, including color, taste, flavor, texture, and acceptability, were selected to establish the evaluation factor set *U* = {*U*_1_, *U*_2_, *U*_3_, *U*_4_, *U*_5_}. Based on the sensory scoring criteria, three quality categories were defined as *V* = {*V*_1_, *V*_2_, *V*_3_}, corresponding to *V*_1_ = “excellent (90–100)”, *V*_2_ = “good (80–89)”, and *V*_3_ = “poor (70–79)”, respectively. To ensure equal contributions from all sensory attributes, identical weights were assigned to the five factors, yielding the weighting vector *X* = {color, taste, flavor, texture, acceptability} = {0.2, 0.2, 0.2, 0.2, 0.2}.

#### 2.9.3. Determination of Fuzzy Matrix

The sensory scores provided by the ten panelists were summarized according to the predefined rating categories. These proportions were used to build a fuzzy relation matrix. To derive the membership degree (*R*) of the five factors for each sample, the tabulated scores were divided by 10, after which the membership values were arranged factor by factor in rows. Based on fuzzy transformation principles, *Y* was defined as the overall evaluation vector covering the products under test, and the fuzzy comprehensive evaluation set was computed as *Y* = *X* × *R*, where *X* represents the weight set and R denotes the fuzzy matrix. Finally, a comprehensive score matrix (*T*) was introduced to process *Y*. In view of the features of sensory scoring, the evaluation level set was defined as *K* = {*K*_1_, *K*_2_, *K*_3_}, with corresponding scores set as *K* = {90, 80, 70}. A sensitivity analysis was performed using four weighting scenarios (equal weighting, flavor-prioritized weighting, texture-prioritized weighting, and exclusion of overall acceptability) to evaluate the robustness of the FCE results.

### 2.10. Statistical Analysis

For GC-IMS analysis, the instrument’s built-in LAV (Laboratory Analytical Viewer) and Gallery Plot tools (G.A.S., Dortmund, Germany) were utilized. LAV was employed for the analysis and annotation of GC-IMS data, in conjunction with the NIST and IMS databases for further in-depth identification, and the Gallery Plot tool was employed to construct the corresponding VOC fingerprint profiles. PCA and PLS-DA were performed using SIMCA 14.1 (MKS Umetrics, Umea, Sweden). Electronic nose data were plotted using Origin 2019 (MicroCal, Northampton, MA, USA). Statistical differences among sample groups were evaluated by one-way ANOVA, followed by Duncan’s multiple range test, using SPSS software (version 19.0; SPSS Inc., Chicago, IL, USA). Results are presented as means ± standard deviations, with statistical significance defined at *p* < 0.05.

## 3. Results and Discussion

### 3.1. Analysis of E-Noses for Different Brands of Sauced Beef

E-nose technology enables rapid characterization of complex food odors through the collective responses of gas sensors to volatile constituents [23]. In the present investigation, an E-nose was employed to distinguish the overall aroma characteristics of the headspace of five sauced beef samples. As illustrated in Figure 1a, the sensor response patterns exhibited broadly similar profiles across the samples, although variations in signal intensity were evident for individual sensors. Sensors W1W, W2W, W5S, W1S, and W2S showed pronounced responses to all five samples, with significant differences observed among the investigated products (*p* < 0.05). These variations may reflect differences in ingredients (e.g., type and amount of soy sauce, spice blends) or manufacturing conditions [9]. By comparison, sensors W3C, W5C, W3S, and W6S exhibited relatively weak responses with largely overlapping patterns among the five samples (*p* > 0.05), suggesting limited variation in the overall odor characteristics detected by these sensors. Compared with sensors W1C, W3C, W5C, W3S, and W6S, sensors W1W, W2W, W5S, W1S, and W2S exhibited more pronounced responses to the VOCs in sauced meat products, suggestive of a stronger contribution from aldehydes, ketones, sulfur-containing compounds, alcohols, and aromatic-related volatiles, consistent with the sensor’s general sensitivity profile. Taken together, the radar profiles provide preliminary evidence of differences in the overall volatile characteristics of the five commercial sauced beef products.

PCA is an unsupervised multivariate analysis method commonly used for preliminary data exploration. To explore variations in overall aroma profiles among the five commercial sauced beef samples, PCA was applied to the E-nose response values (Figure 1b). PC1 and PC2 explained 66.5% and 24.0% of the total variance, respectively, yielding a cumulative contribution of 90.50%. This indicates that the two-dimensional PCA representation retained most of the variation in the sensor response data. Samples B4 and B5 were separated from the other three groups, yet all samples were clustered in the central region with overlapping confidence ellipses. The pattern thus reveals partial similarity in VOCs of the five samples, indicating a common aroma basis along with subtle product-associated variations. The characteristic flavor of spiced braised meat products may be related to variations in seasonings and spices, with additional complexity arising from chemical reactions during processing [3]. Nevertheless, the E-nose primarily provides an overall response pattern and cannot reliably identify or quantify individual VOCs [24]. Because the E-nose findings, which pointed to overlapping and potentially distinct aroma characteristics, GC-IMS was employed to further characterize the VOCs potentially contributing to the observed odor differences.

### 3.2. Analysis of VOCs for Different Brands of Sauced Beef

#### 3.2.1. GC-IMS Analysis

GC-IMS couples ion mobility detection with chromatographic separation, thereby allowing complex mixtures of VOCs to be characterized. The GC-IMS fingerprints contain the raw information of all compounds and can be used to analyze the VOCs in complex samples.

Figure 2a shows the GC-IMS three-dimensional spectra of VOCs in five brands of sauced beef. In these spectra, each dot corresponds to a compound, and the color represents the signal intensity. The depth of the dot color directly reflects the content of the substance: a darker color indicates a higher content. Analysis of the GC-IMS spectra demonstrated noticeable variations in VOC profiles across the investigated samples.

To further examine these variations, differential GC-IMS maps were generated using B1 as the reference sample (Figure 2b). In these plots, variations in signal color illustrate the relative differences between each product and B1. Red dots indicate stronger signals than those of the reference, whereas white dots represent weaker responses. The signal variations reveal differences in VOCs among the five samples, facilitating further compound characterization.

Fingerprint profiles were constructed to further characterize VOCs and investigate their differences among samples. Figure 2c illustrates the distinctive volatile fingerprint patterns obtained for the five sauced beef products. In Figure 2c, each row represents the volatile characteristics of one sample, while each column corresponds to the signal peak intensity of a specific volatile compound across different samples. Brighter colors indicate higher contents, while darker colors indicate lower contents. This visualization enables an intuitive comparison of differences in the same VOCs among sample groups. Although the types of VOCs detected in the different sauced beef samples were similar, significant differences in the relative contents of some compounds were observed, forming common peak regions and characteristic peak regions. By analyzing the VOCs in these regions, the flavor characteristics of different brands of sauced beef can be understood.

As shown in Figure 2c, five regions (A–E) in the GC-IMS fingerprint correspond to characteristic volatile clusters. Region A, dominated by acids, esters, and sulfur-containing compounds, is most enriched in sample B1, imparting sweetness, fresh fruity, woody, cheesy, and sulfurous notes. Region B, rich in alcohols, terpenes, and disulfides, characterizes B2, contributing herbal, earthy fennel, fresh fruity, alcoholic, buttery, nutty, and garlic-like odors. Region C, mainly comprising terpenes, pyrazines, and ketones, peaks in B3, yielding fruity, floral, herbal, mushroom, creamy, nutty, and roasted beef flavors. Region D, dominated by aldehydes and 1,8-Cineole, is characteristic of B4, giving fruity, grassy, fatty, nutty, and refreshing minty notes. Region E, consisting mainly of aldehydes and ketones, is most abundant in B5, producing balsamic, banana-like, grassy, fruity, and fatty odors. Collectively, the VOCs enriched in each brand differ in both identity and concentration, underpinning the unique flavor profile of each sauced beef product.

Qualitative analysis of VOCs in different brands of sauced beef was performed by GC-IMS. The contents and compositions of VOCs in the five samples are presented in Table 4 and Table 5. A total of 73 VOCs were tentatively identified. The total numbers of volatile substances in samples B1-B5 were 58, 63, 64, 56, and 52, respectively. These belonged to nine major categories: hydrocarbons, alcohols, aldehydes, ketones, esters, acids, sulfur-containing compounds, nitrogen-containing compounds, and others. Among the 73 tentatively identified compounds, the monomers and dimers of 25 compounds were included. The types and contents of VOCs varied markedly among different brands. Ketones (30.01–32.33%), alcohols (13.55–21.04%), and aldehydes (12.43–30.35%) accounted for relatively high proportions, followed by esters (5.47–15.76%).

B3 exhibited the highest diversity of VOCs, while B5 showed the lowest (Table 4), potentially reflecting differences in spice complexity and processing intensity [3]. Ketones accounted for >30% of the total volatiles in all five sauced beef samples, with the maximum content (32.33%) observed in sample B1. Meanwhile, alcohols showed the highest relative content (21.04%) in sample B2. The aldehyde contents in samples B4 and B5 exceeded 30%, nearly twice that in B1-B3 (approximately 12–17%). Aldehydes are mostly thermal degradation products of lipids, suggesting more extensive lipid oxidation in these products [25]. Previous studies also found that aldehydes accounted for the highest percentage of flavor compounds in various cooked meat products [26]. These differences may be related to processing technology and spice ratios [3]. Sun D et al. [9] found that the acetone content of Yanbian yellow beef was higher than that of other beef sources, indicating that beef breed also affects the types of flavor compounds. Ketones, aldehydes, alcohols, and esters represent the predominant classes of volatiles in sauced beef [9]. Together, these volatile constituents contribute to the distinctive flavor profile of sauced beef.

#### 3.2.2. PLS-DA Model Analysis

PLS-DA employs supervised dimensionality and facilitates visualization, discrimination, and prediction of complex datasets. To more intuitively reveal the differences in VOCs among the five sauced beef samples and to screen key VOCs, supervised PLS-DA modeling was performed. As illustrated in Figure 3, the PLS-DA model achieved R^2^x, R^2^y, and Q^2^ values of 0.983, 0.995, and 0.990, all exceeding 0.5, suggesting a strong fit to the analyzed dataset and high cross-validation performance. In Figure 3a, the distribution of samples in each group is clearly separated. The biplot (Figure 3b) showed good separation among the five sample groups, with the compounds relatively dispersed. Most VOCs and aroma attributes fell between the inner and outer ellipses, indicating good explanatory power of the model. Permutation tests (*n* = 200) were performed to verify whether the model was overfitted (Figure 3c). The obtained R^2^ values were (0.0, 0.0689) and Q^2^ values were (0.0, −0.674). All R^2^ values were positioned above the horizontal axis. The negative Q^2^ regression line suggests that the model is reliable and has high cross-validated predictive performance within the investigated dataset [27].

The relative importance of individual VOCs in distinguishing the sample groups was evaluated using VIP scores derived from the PLS-DA model. A higher VIP value indicates a greater contribution to discriminating the sample groups. Using VIP > 1 as a threshold, increasing VIP values indicated more pronounced differences in VOCs among brands. A total of 27 significant VOCs were screened, as shown in Figure 3d. These included six ketones, five aldehydes, five alcohols, three esters, three hydrocarbons, two sulfur-containing compounds, one nitrogen-containing compound, and two other compounds.

#### 3.2.3. Analysis of Potential Aroma-Active Differential Compounds

The distinctive flavor profiles of sauced meat products arise from both exogenous seasonings and spices and endogenous reactions during processing [3], producing VOCs of diverse chemical classes that determine the product’s sensory properties [28]. The aroma contribution of individual VOCs is influenced by their abundance and corresponding odor detection thresholds; the *OAV* evaluates aroma impact more reliably than relative content alone. The flavor-influencing factors mainly include relative content and odor threshold. Volatiles with *OAV* ≥ 1 have aroma impact more reliably than relative content alone [29]. For the 27 differential VOCs with VIP > 1 detected by GC-IMS, *OAVs* were calculated. Of the 27 differential compounds (VIP > 1) detected by GC-IMS, 14 met the combined *OAV* ≥ 1 and VIP > 1 criteria (Table 6).

As shown in Figure 4, the five samples shared nine common potential aroma-active compounds, including 1-Hexanol, 1-Octen-3-ol, (R/S)-linalool, 2-Methylpropanal, 3-Methylbutanal, (E)-2-hexenal, 1-Nonanal, 2-Heptanone, and 2-Methyl-2-hepten-6-one—forming the common aroma foundation of sauced beef. These alcohols, arising mainly from aldehyde reduction and lipid oxidation [30], contribute fresh-green (1-Hexanol [31]), sweet floral, rose-like notes ((R/S)-linalool [32]), and mushroom, herbal (1-Octen-3-ol [32]) notes. The aldehydes, generally with low thresholds [3], comprise Strecker-derived 2-Methylpropanal and 3-Methylbutanal (malty, fruity [4]) and lipid-oxidation products (E)-2-hexenal and 1-Nonanal (oily, fatty [4,33]). The ketones 2-Heptanone and 2-Methyl-2-hepten-6-one provide a creamy, fruity background [34]; the latter enhances richness and reduces greasiness [35], while the former adds fermented complexity at low concentration but yields pungent off-odors at high concentration [36]. This shared profile agrees with Sun D et al. [9] on Yanbian-style sauced beef.

Beyond this common base, each brand displayed a distinct volatile signature. B2 contained the most potential aroma-active compounds (12 of 14), including two unique compounds—(E)-3-hexen-1-ol (green-leaf aroma, from plant spices [3]) and Acetic acid hexyl ester (fruity, sweet [37]); the latter, abundant in vacuum-cooled sauced beef [37], suggests a distinctive processing technology for B2. B3 was distinguished by 2-Methyl-3-furanthiol, a Strecker-derived thiol conferring roasted-meat and coffee-like aromas [38], in line with its roasted-beef, creamy profile. B4 was characterized by Isomenthone, a spice-derived terpenoid with a cooling, minty note [39], complementing its aldehyde-driven fatty and grassy character. B1 and B5 both contained 2-Pentylfuran, a linoleic-acid oxidation marker with nutty, caramel, meaty and butter-like notes [25], alongside the common aldehydes and ketones; B1 was further marked by cheesy and sulfurous tones (Figure 2c, Region A), whereas B5 leaned toward balsamic, banana-like and fruity odors (Figure 2c, Region E).

Collectively, alcohols (five, including one thiol) dominated the potential-aroma set, followed by aldehydes (four), ketones (three), esters (one), and other compounds (one). The brand-to-brand differences reflect processing technology, raw-material origin, and spice/seasoning proportions: spices introduce characteristic compounds and promote flavor reactions, while salt and sugar participate in lipid hydrolysis and the Maillard reaction [3]; soy sauce provides intense umami and saltiness via free amino acids, nucleotides, umami peptides, and sodium ions; its fermentation products can also synergize with Maillard-derived heterocyclic compounds [40]. During curing, stewing, packaging and sterilization, integrated Maillard–Strecker reactions, lipid and protein oxidation, and nucleic-acid degradation further layer the flavor [3], providing a theoretical basis for flavor regulation of sauced meat products.

### 3.3. Texture Profile Analysis

Texture properties are important indicators for determining overall product acceptability. Within a certain range, changes in texture properties are mainly related to the composition, tissue structure, and cut of the food itself. The texture changes in sauced meat products are usually influenced by protein denaturation, lipid oxidative degradation, moisture content, and other factors [41]. As shown in Table 7, differences in textural properties were observed among the five samples (*p* < 0.05). Compared with the other four groups, sample B1 exhibited significantly higher hardness, chewiness, and gumminess (*p* < 0.05). In contrast, cohesiveness was highest in sample B2 and lowest in sample B3, while no significant differences were observed between B1 and B4 or B5. For springiness, B1 showed the highest numerical value, but it did not differ significantly from B2 or B4 (*p* > 0.05). These variations in textural properties may be associated with differences in raw materials (e.g., breed, cut, or age) and processing conditions (e.g., stewing time and temperature) [41]. However, further investigation is required to confirm these potential contributing factors. The observed textural variations may also be related to differences in sensory evaluation scores among the five samples.

### 3.4. Result of Sensory Evaluation

Using the data presented in Table 3, the fuzzy evaluation matrix (*R*) was composed of the proportions of assessors assigning ratings of the color, taste, flavor, texture, acceptability of samples in the grade of “excellent”, “good”, and “poor”. The corresponding rating distributions for each attribute are summarized in Table 8.

$$R_{1}=\left|\begin{array}{c} \begin{array}{c} \begin{array}{cc} 0.1 & \begin{array}{cc} 0.9 & 0 \end{array} \end{array}\\ \begin{array}{cc} 0.2 & \begin{array}{cc} 0.8 & 0 \end{array} \end{array}\\ \begin{array}{cc} 0.4 & \begin{array}{cc} 0.6 & 0 \end{array} \end{array} \end{array}\\ \begin{array}{cc} 0.3 & \begin{array}{cc} 0.7 & 0 \end{array} \end{array}\\ \begin{array}{cc} 0.5 & \begin{array}{cc} 0.5 & 0 \end{array} \end{array} \end{array}\right|$$

$$R_{2}=\left|\begin{array}{c} \begin{array}{c} \begin{array}{cc} 0.8 & \begin{array}{cc} 0.2 & 0 \end{array} \end{array}\\ \begin{array}{cc} 0.6 & \begin{array}{cc} 0.4 & 0 \end{array} \end{array}\\ \begin{array}{cc} 0.6 & \begin{array}{cc} 0.4 & 0 \end{array} \end{array} \end{array}\\ \begin{array}{cc} 0.7 & \begin{array}{cc} 0.3 & 0 \end{array} \end{array}\\ \begin{array}{cc} 0.5 & \begin{array}{cc} 0.5 & 0 \end{array} \end{array} \end{array}\right|$$

$$R_{3}=\left|\begin{array}{c} \begin{array}{c} \begin{array}{cc} 0.8 & \begin{array}{cc} 0.2 & 0 \end{array} \end{array}\\ \begin{array}{cc} 0.4 & \begin{array}{cc} 0.6 & 0 \end{array} \end{array}\\ \begin{array}{cc} 0.3 & \begin{array}{cc} 0.7 & 0 \end{array} \end{array} \end{array}\\ \begin{array}{cc} 0.7 & \begin{array}{cc} 0.3 & 0 \end{array} \end{array}\\ \begin{array}{cc} 0.5 & \begin{array}{cc} 0.5 & 0 \end{array} \end{array} \end{array}\right|$$

$$R_{4}=\left|\begin{array}{c} \begin{array}{c} \begin{array}{cc} 0.3 & \begin{array}{cc} 0.7 & 0 \end{array} \end{array}\\ \begin{array}{cc} 0.9 & \begin{array}{cc} 0.1 & 0 \end{array} \end{array}\\ \begin{array}{cc} 1.0 & \begin{array}{cc} 0.0 & 0 \end{array} \end{array} \end{array}\\ \begin{array}{cc} 0.1 & \begin{array}{cc} 0.9 & 0 \end{array} \end{array}\\ \begin{array}{cc} 0.5 & \begin{array}{cc} 0.5 & 0 \end{array} \end{array} \end{array}\right|$$

$$R_{5}=\left|\begin{array}{c} \begin{array}{c} \begin{array}{cc} 0.6 & \begin{array}{cc} 0.4 & 0 \end{array} \end{array}\\ \begin{array}{cc} 0.4 & \begin{array}{cc} 0.6 & 0 \end{array} \end{array}\\ \begin{array}{cc} 0.8 & \begin{array}{cc} 0.2 & 0 \end{array} \end{array} \end{array}\\ \begin{array}{cc} 0.5 & \begin{array}{cc} 0.5 & 0 \end{array} \end{array}\\ \begin{array}{cc} 0.4 & \begin{array}{cc} 0.6 & 0 \end{array} \end{array} \end{array}\right|$$

The overall sensory evaluation vectors were obtained by multiplying the attribute weighting vector (*X*) by the corresponding evaluation matrix (*R*), following *Y* = *X* × *R*. For sample B1, the calculation was performed as follows:

$$Y_{1}\;=\;X\times R_{1}=\left|\begin{array}{ccc} 0.2 & 0.2 & \begin{array}{ccc} 0.2 & 0.2 & 0.2 \end{array} \end{array}\right|\times \left|\begin{array}{c} \begin{array}{c} \begin{array}{cc} 0.1 & \begin{array}{cc} 0.9 & 0 \end{array} \end{array}\\ \begin{array}{cc} 0.2 & \begin{array}{cc} 0.8 & 0 \end{array} \end{array}\\ \begin{array}{cc} 0.4 & \begin{array}{cc} 0.6 & 0 \end{array} \end{array} \end{array}\\ \begin{array}{cc} 0.3 & \begin{array}{cc} 0.7 & 0 \end{array} \end{array}\\ \begin{array}{cc} 0.5 & \begin{array}{cc} 0.5 & 0 \end{array} \end{array} \end{array}\right|=\{0.30,0.70,0\}$$

Applying the same procedure to the other products yielded *Y*_2_ = {0.64, 0.36, 0}, *Y*_3_ = {0.54, 0.46, 0}, *Y*_4_ = {0.56, 0.44, 0}, and *Y*_5_ = {0.54, 0.46, 0}. The FCE score was determined by T = Y × K with the evaluation set *K* = {90, 80, 70}, representing the excellent, good, and poor categories, respectively. Taking B1 as an example, the comprehensive score was calculated as

$$T_{1}=Y_{1}\times K=\left|\begin{array}{ccc} 0.30 & 0.70 & 0 \end{array}\right|\times \left|\begin{array}{c} 90\\ 80\\ 70 \end{array}\right|=83.0$$

Similarly, *T*_2_ = 86.4, *T*_3_ = 85.4, *T*_4_ = 85.6, and *T*_5_ = 85.4. The calculation showed that the evaluation scores of the five sauced beef samples were all greater than 80 points, belonging to the good level. Among the investigated samples, B2 achieved the highest overall sensory rating, whereas B1 obtained the lowest. The sensory scores of the five samples were ranked from high to low as B2 > B4 > B3 = B5 > B1. These findings indicate differences in the comprehensive sensory scores assigned by the trained assessors, although they should not be interpreted as representing consumer preferences.

To assess the robustness of the FCE, a sensitivity analysis was conducted using alternative weighting schemes and excluding overall acceptability. The recalculated comprehensive scores and rankings are presented in Supplementary Table S1. Although some variations in sample rankings were observed, B2 ranked first under three of the four weighting scenarios, while B4 ranked first when flavor was prioritized. These findings suggest that the evaluation results were relatively stable under certain weighting adjustments, although some sensitivity to the weighting schemes remained.

### 3.5. Correlation Between Sensory Score and Potential Aroma-Active Differential Compounds

To enhance the overall efficiency of sensory evaluation and correlate it with potential aroma-active compounds, this work aimed to explore the possible association between sensory scores and the levels of differential VOCs identified by GC-IMS. To explore potential associations between sensory attributes and aroma-active constituents, Pearson’s correlation coefficients were calculated using the ratings for color, taste, flavor, texture, and acceptability along with the measured levels of VOCs exhibiting *OAV* > 1 (Figure 5). The aroma scores were positively associated with 3-(Methylsulfanyl)propanal, (Z)-2-Pentenal, (E)-2-Butenal and Isomenthone, whereas negative associations were observed with 3-Methyl butanal, Dimethyl sulfide, 2-Methyl-1-propyl acetate, (R/S)-linalool, Acetic acid ethyl ester, 2-Butanone-3-hydroxy, Allyl sulfide, alpha-Pinene, 2-Pentanone, Propannoic acid, Acetic acid and 2-Propanone. Similarly, flavor scores showed positive associations with 3-(Methylsulfanyl)propanal, 1-Nonanal, Benzaldehyde, (Z)-2-Pentenal, 2-Methylpropanal, Heptaldehyde and 1-Penten-3-ol, while negative associations were observed with 2-Methyl-3-furanthiol, (R/S)-linalool, 2-Butanone-3-hydroxy, alpha-Terpinolene and alpha-Pinene. Correlation analysis revealed potential associations between certain VOCs and sensory attributes across the five products. However, given the limited number of observations (*n* = 5) and the potential risk of false-positive findings due to multiple comparisons, these correlations should be interpreted as preliminary associations rather than definitive relationships.

In general, compounds showing positive correlations tended to contribute green, fruity, fatty, nutty and cool notes, whereas those with negative correlations were characterized by fresh, sweet fruity, wine, woody, butter, sulfur and fatty notes. Notably, higher linalool concentrations may contribute to undesirable pungent notes [42], potentially explaining some of the observed negative associations. From a quality evaluation perspective, the identified potential aroma-active compounds, particularly the positively associated compounds (3-(Methylsulfanyl)propanal, (Z)-2-Pentenal), may offer preliminary indicators for assessing product consistency. The results indicated a certain correlation between sensory score and potential aroma-active differential compounds, implying that these factors can be integrated to characterize sauced beef more effectively. Nonetheless, a Pearson correlation by itself does not prove any causal relationship between VOCs and sensory attributes. Whether these associations are of practical use still needs confirmation through sensory recombination experiments (e.g., omission tests) or model system studies.

## 4. Conclusions

In this investigation, VOC profiles across five commercially available sauced beef products were examined using an E-nose, GC-IMS, and multivariate chemometric analysis. TPA and FCE were integrated to assess textural and sensory quality. The analysis revealed statistically significant associations between the evaluated sensory characteristics and instrument-derived measurements. E-nose showed similar odor profiles across brands of sauced beef, though the odor response intensities differed. Sensors W1W, W2W, W5S, W1S, and W2S responded markedly to the samples. Nevertheless, the five samples clustered closely in the PCA and were not well discriminated. To further clarify the volatile differences among brands of sauced beef, the samples were analyzed by GC-IMS. GC-IMS detected 73 VOCs, with ketones, alcohols, aldehydes, esters, and hydrocarbons as the predominant classes. PLS-DA modeling screened 27 VOCs with VIP > 1.0. From the intersection of *OAV* > 1 and VIP > 1, 14 potential aroma-active differential compounds were identified, of which 9 were common to all five samples: 1-Hexanol, 1-Octen-3-ol, (R/S)-linalool, 2-Methylpropanal, 3-Methylbutanal, (E)-2-hexenal, 1-Nonanal, 2-Heptanone, and 2-Methyl-2-hepten-6-one. These nine substances are proposed as the core characteristic flavor compounds of commercial sauced beef. Characteristic compounds included (E)-3-hexen-1-ol for sample B2 and 2-Methyl-3-furanthiol for sample B3. Marked differences in TPA were observed among the five sauced beef samples. Sample B1 showed higher values for hardness, chewiness, and gumminess than the other four samples (*p* < 0.05). Using equal weights of 0.2 for each sensory attribute, FCE ranked overall sensory quality as B2 > B4 > B3 = B5 > B1, indicating that samples B2 and B4 were superior to samples B3, B5, and B1 based on fuzzy mathematical evaluation. Correlation analysis revealed that taste and flavor attributes were significantly positively correlated with the concentrations of 3-(Methylsulfanyl)propanal and (Z)-2-pentenal (*p* < 0.05). In contrast, (R/S)-linalool, 2-Butanone-3-hydroxy, and alpha-Pinene were negatively correlated with these sensory attributes (*p* < 0.05).

Collectively, the five commercial sauced beef products exhibited differences in volatile profiles, textural properties, and sensory characteristics. These findings provide preliminary insights into product-specific quality characteristics and suggest potential discriminatory compounds. Integrating instrumental flavor profiling with fuzzy sensory evaluation provided preliminary insights into product differentiation and potential quality-related compounds. This work offers a scientific reference for product differentiation and supports the development of more diversified, high-quality sauced beef products. Future studies involving larger sample sets and independent validation are needed to clarify the effects of raw materials and processing conditions and support consumer-oriented product development.

## Figures and Tables

**Figure 1:**
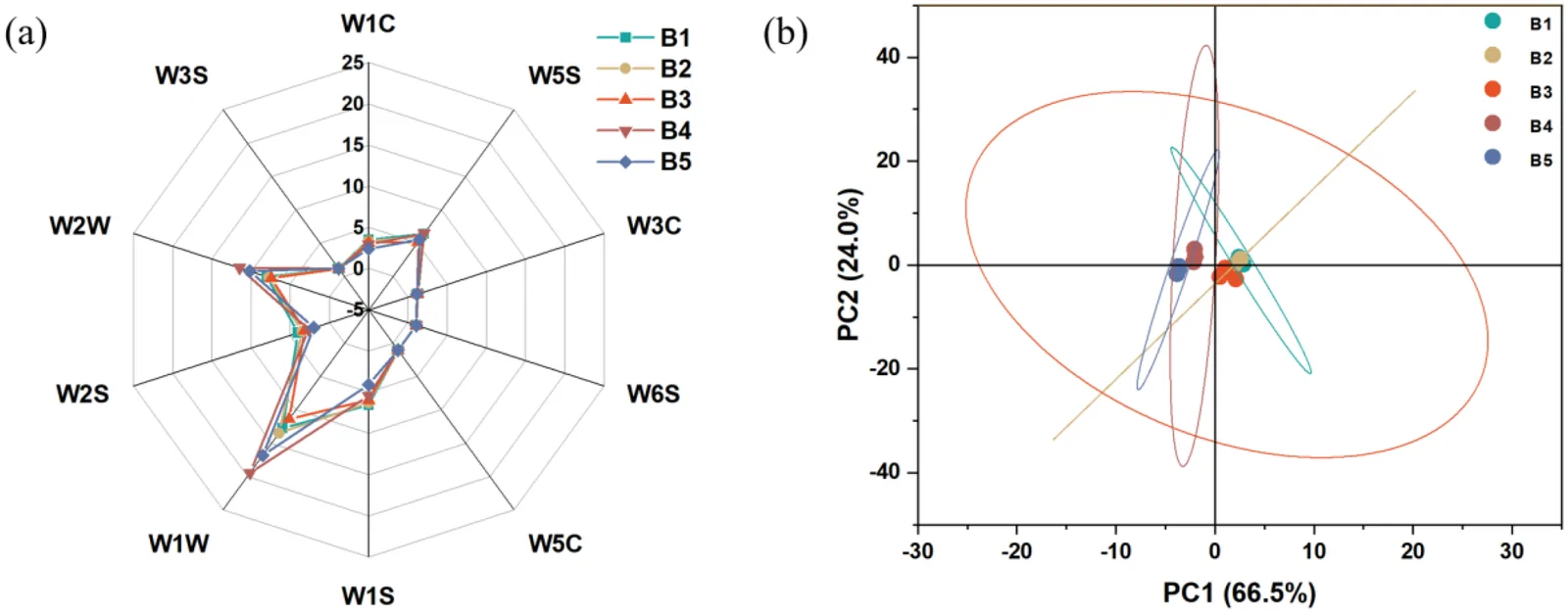
Radar diagram of the E-nose response data (**a**) and PCA score plot (**b**) of sauced beef.

**Figure 2:**
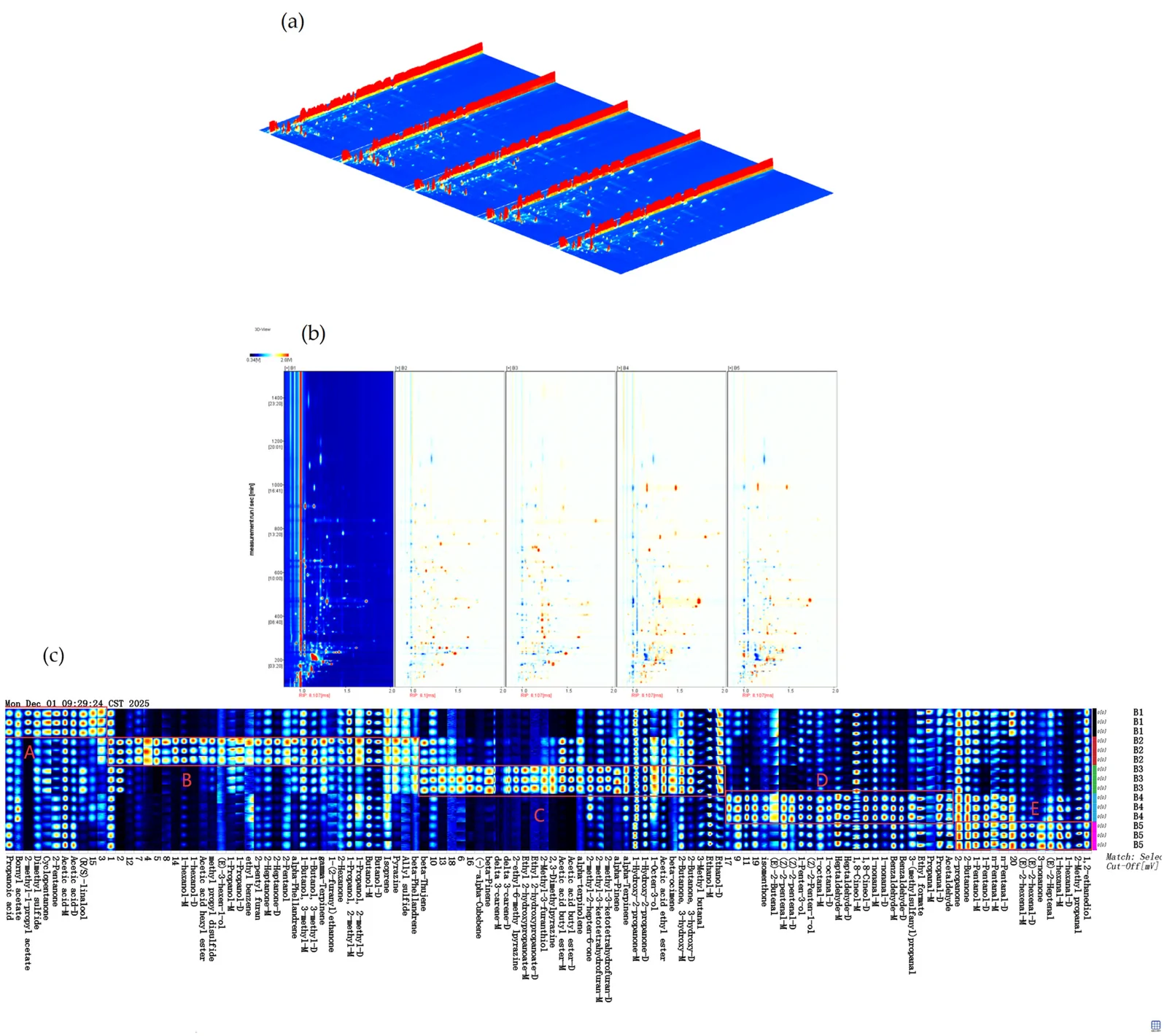
GC-IMS analysis of VOCs of five sauced beef samples: three-dimensional (3D) topographic (**a**), difference spectrum (**b**), fingerprint spectra (**c**).

**Figure 3:**
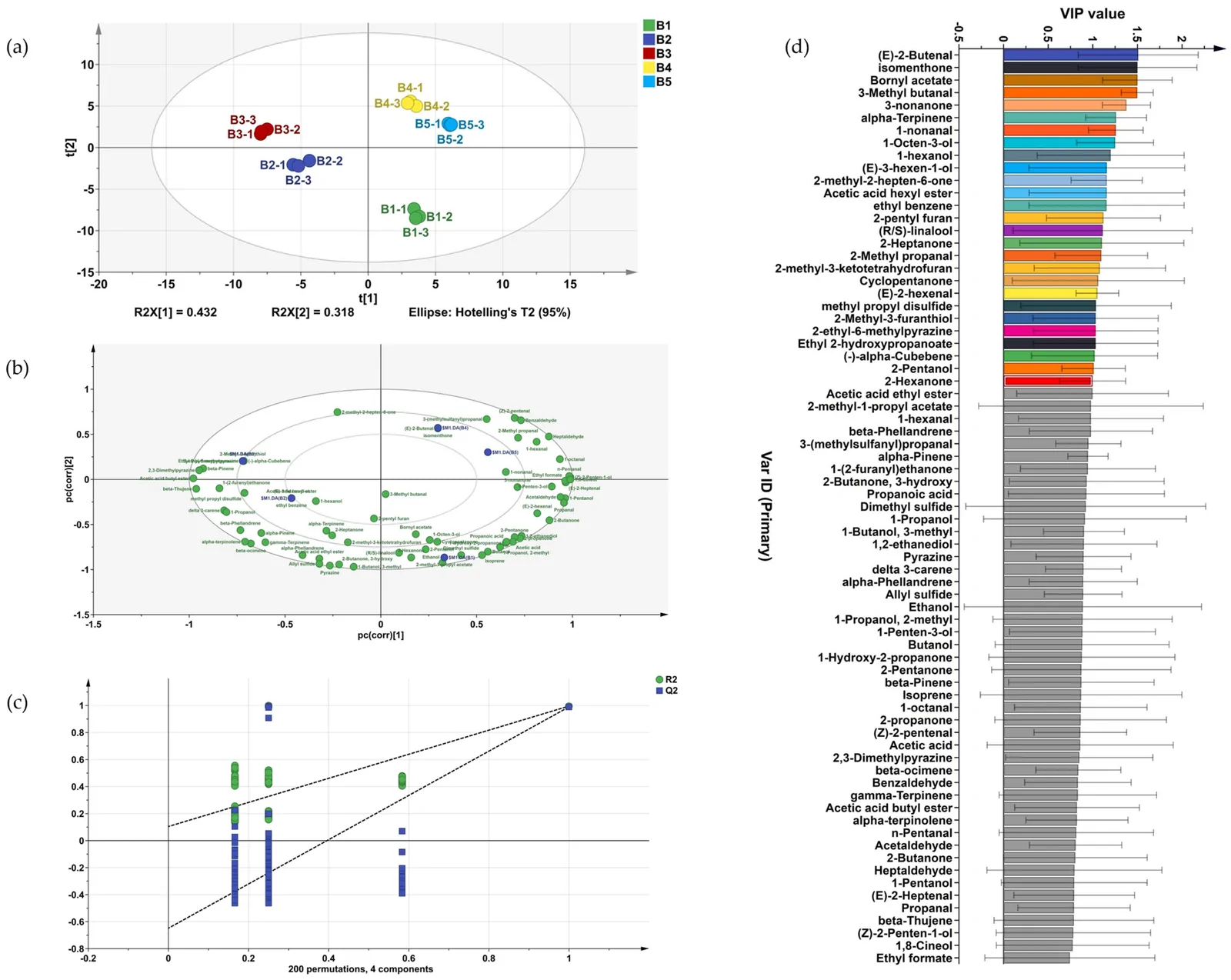
PLS-DA (**a**), biplot (**b**), permutation test (**c**), and VIP value (**d**) of VOCs of five sauced beef samples with PLS-DA model.

**Figure 4:**
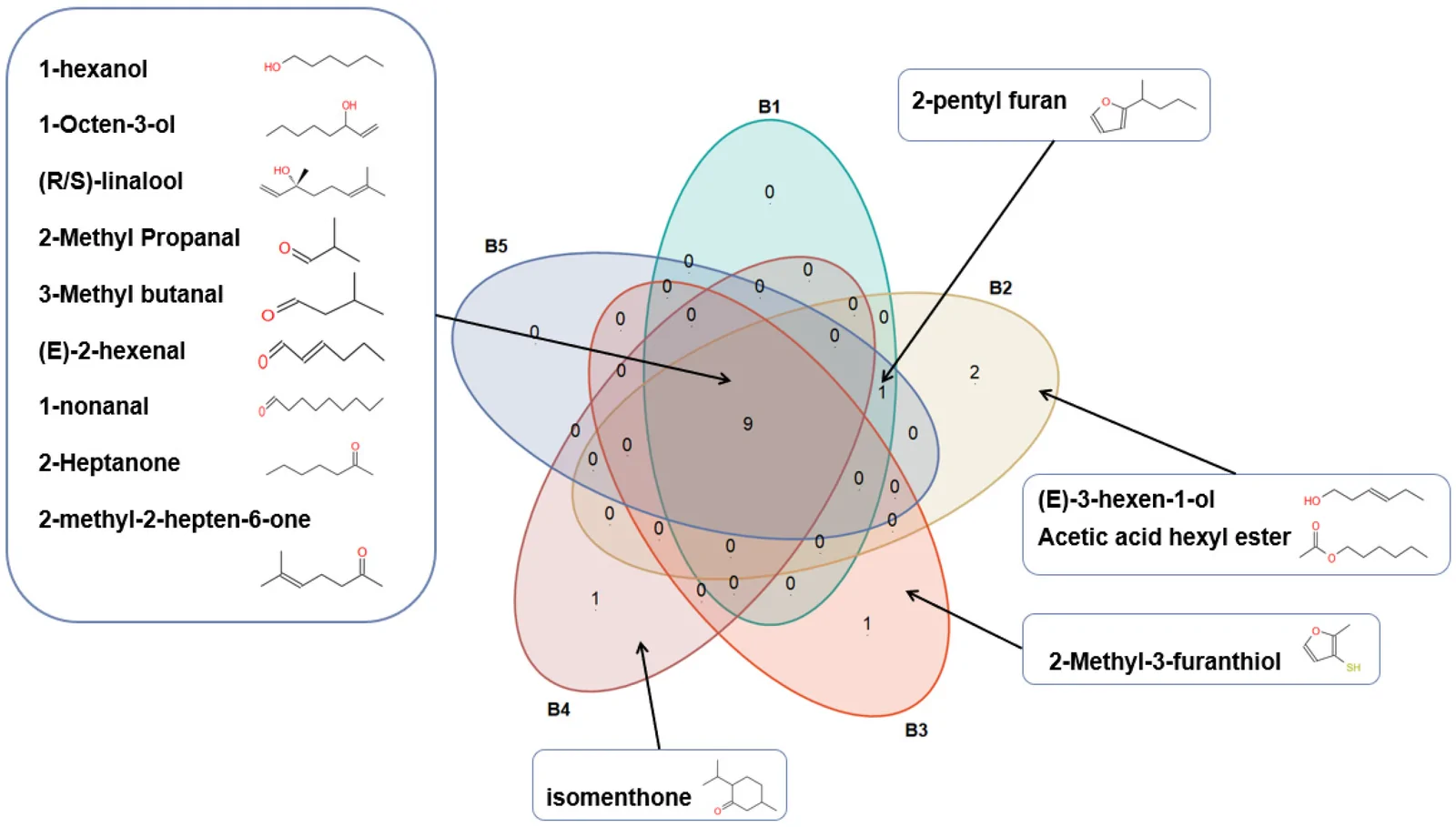
Venn diagram of volatile compound profiles of five different beef samples. Note: The numbers inside the diagram represent the quantity of volatile compounds in each.

**Figure 5:**
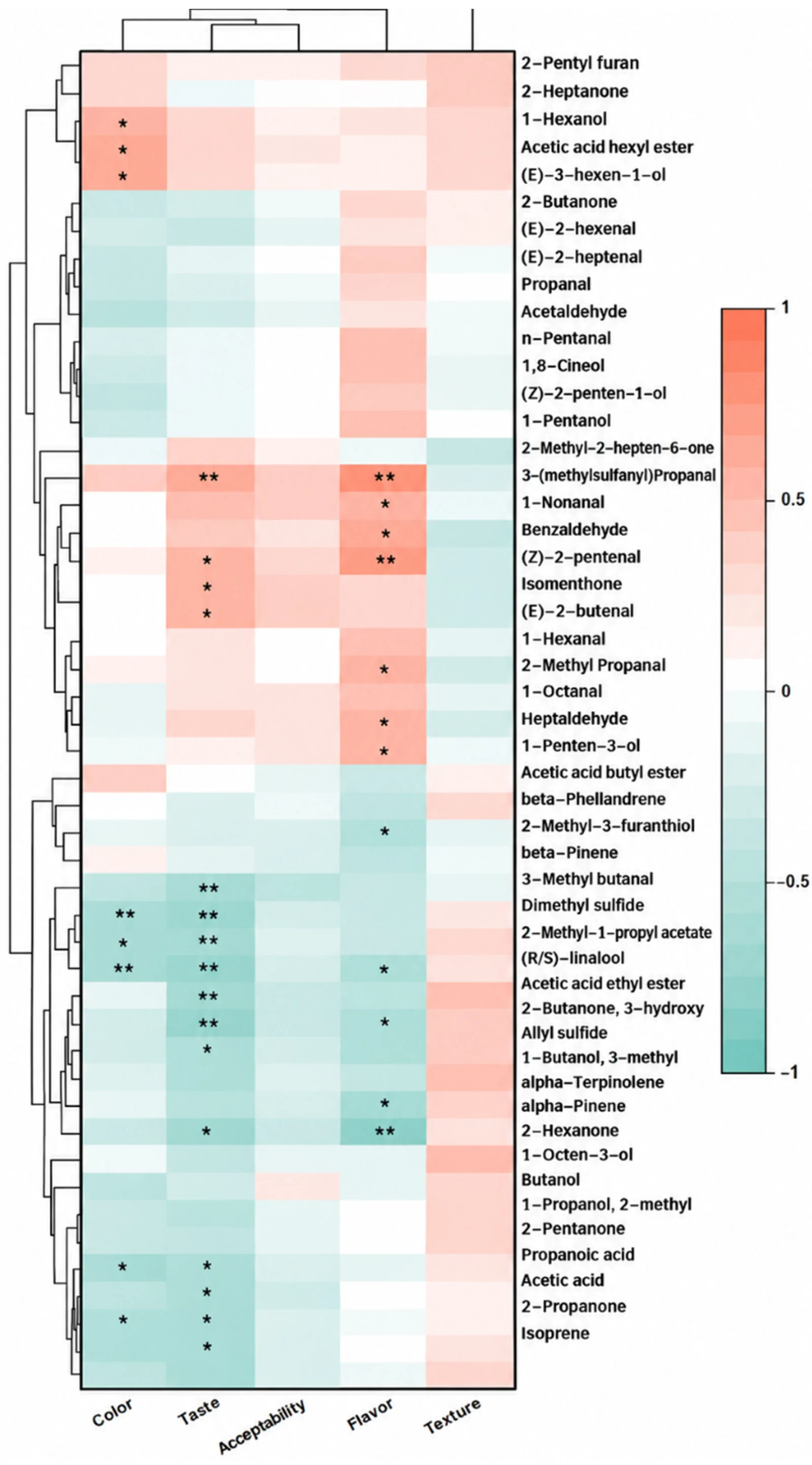
Pearson correlation map between the relative content of flavor substances in sauced beef and their and sensory evaluation. Note: * indicates *p* < 0.05, and ** indicates *p* < 0.01. Positive (0 < R < 1) and negative (−1 < R < 0) correlations are denoted by red and blue colors, respectively. Given the limited sample size (*n* = 5), these correlations should be interpreted as exploratory associations.

**Table 1:** Detailed information of the different brands of sauced beef.

Sample Number	Name	Execution Standard	Principal Ingredients
B1	Sauced beef	GB/T 23586 [20]	Beef, rock sugar, salt, brewed soy sauce, oyster sauce, soybean paste, spices
B2	Original-Cut sauced beef	GB 2726 [21]	Beef, salt, monosodium glutamate, brewed soy sauce (including caramel color), compound seasoning, spices
B3	Light sauced beef shank	GB/T 23586 [20]	Beef shank, soybean paste, salt, compound seasoning, spices, brewed soy sauce (including caramel color), dried tangerine peel, angelica dahurica
B4	Sauced beef	GB/T 23586 [20]	Beef, soybean paste, salt, compound seasoning, brewed soy sauce (including caramel color), spices
B5	Sauced beef	GB/T 23586 [20]	Beef shank, sweet soybean paste, soybean paste, salt, compound seasoning, brewed soy sauce, beef tallow, spices

**Table 2:** E-nose sensor array names and corresponding features.

Sensor Number	Sensors Name	Performance Description
1	W1C	Sensitive to Aromatic compounds
2	W5S	Sensitive to nitrogen oxides
3	W3C	Sensitive to ammoniaand aromatic components
4	W6S	Sensitive to hydrides
5	W5C	Sensitive to olefin, aromatic components and polar molecules
6	W1S	Sensitive to alkanes
7	W1W	Sensitive to sulfides
8	W2S	Sensitive to alcohols and some aromatic compounds
9	W2W	Sensitive to aromatic components and organic sulfides
10	W3S	Sensitive to aliphatic series

**Table 3:** Sensory evaluation criteria of different sauced beef samples.

Items	*V*_1_	*V*_2_	*V*_3_
Color	Uniform cross-section color, reddish brown or pinkish red, natural and oily.	Relatively uniform cross-section color, dull, excessively dark or light.	Non-uniform cross-section color, lusterless, dark and dull.
Taste	Well-balanced salty and umami flavor, rich meaty taste with sweet aftertaste.	Well-balanced salty and umami flavor, relatively rich meaty taste with slight aftertaste.	Overly salty, bland, no meaty taste, no aftertaste or with off-flavors.
Flavor	Rich and harmonious spice sauce aroma, pure meaty aroma, no off-odors.	Relatively harmonious spice aroma, indistinct meaty aroma, slight off-odors.	Indistinct spice aroma, weak meaty aroma, with off-odors.
Texture	Compact and uniform structure, moderate hardness and elasticity.	Relatively compact structure, relatively uniform texture, weak elasticity.	Loose structure, rough texture, tough or mushy mouthfeel.
Acceptability	Highly acceptable, very favorable overall impression.	Relatively acceptable, generally favorable impression.	Marginally acceptable, barely favorable impression.

Note: *V*_1_ = excellent (90–100); *V*_2_ = good (80–89); and *V*_3_ = poor (70–79).

**Table 4:** Types and relative contents of the VOCs of five sauced beef samples tentatively identified and quantified using GC-IMS.

Types of Compounds	B1		B2		B3		B4		B5	
	Number	Relative Content (%)	Number	Relative Content (%)	Number	Relative Content (%)	Number	Relative Content (%)	Number	Relative Content (%)
Hydrocarbons	10	5.55	12	8.95	12	12.21	8	3.27	6	1.91
Alcohols	13	19.91	14	21.04	13	13.55	13	16.53	13	15.75
Aldehydes	13	16.64	12	13.18	12	12.43	14	30.34	13	30.35
Ketones	10	32.33	9	30.12	10	30.93	10	30.01	10	30.69
Esters	4	9.27	5	13.24	5	15.76	3	5.47	2	6.06
Acids	2	5.88	2	4.09	2	3.71	2	4.04	2	5.2
S-containings	2	2.28	4	2.05	4	1.94	2	1.44	2	1.49
N-containings	1	0.82	2	1.13	3	1.81	1	0.24	1	0.21
Others	3	7.32	3	6.19	3	7.66	3	8.66	3	8.34
Total	58	100.00	63	100.00	64	100.00	56	100.00	52	100.00

**Table 5:** The volatile flavor composition of different brands of five sauced beef samples.

Count	Compound	CAS #	Formula	MW	RI	Rt [s]	Dt [a.u.]	Semi-Quantitative Content/(μg/kg)				
								B1	B2	B3	B4	B5
Hydrocarbons (13)												
1	Isoprene	C78795	C5H8	68.1	681.6	111.273	1.06416	557.86 ± 23.49 ^a^	337.59 ± 9.21 ^b^	217.69 ± 3.28 ^d^	295.54 ± 5.04 ^c^	341.57 ± 4.62 ^b^
2	alpha-Pinene	C80568	C10H16	136	1037.1	265.331	1.22234	1016.13 ± 34.24 ^a^	668.42 ± 68.10 ^b^	1138.42 ± 96.74 ^a^	314.94 ± 71.75 ^c^	249.42 ± 53.86 ^c^
3	beta-Pinene	C127913	C10H16	136	1108.5	333.877	1.22196	ND	293.71 ± 41.55 ^b^	731.43 ± 35.19 ^a^	ND	ND
4	beta-Thujene	C28634891	C10H16	136	1123.1	351.689	1.22318	220.46 ± 23.39 ^b^	1255.15 ± 222.37 ^a^	1153.34 ± 104.19 ^a^	136.87 ± 32.97 ^c^	ND
5	Ethyl benzene	C100414	C8H10	106	1161	402.777	1.0746	ND	90.25 ± 6.68	ND	ND	ND
6	delta 3-Carene	C13466789	C10H16	136	1163.2	406.034	1.22287	672.70 ± 8.46 ^b^	627.66 ± 90.61 ^b^	1164.15 ± 54.87 ^a^	187.49 ± 60.55 ^c^	193.00 ± 76.06 ^c^
7	alpha-Phellandrene	C99832	C10H16	136	1178.4	428.682	1.22287	780.15 ± 13.99 ^a^	830.28 ± 167.63 ^a^	440.41 ± 55.05 ^b^	197.33 ± 55.69 ^c^	184.1 ± 33.33 ^c^
8	alpha-Terpinene	C99865	C10H16	136	1197.4	454.869	1.22062	1036.91 ± 28.48 ^a^	719.25 ± 88.73 ^b^	882.82 ± 3.50 ^ab^	769.53 ± 189.64 ^b^	451.37 ± 27.27 ^c^
9	beta-Phellandrene	C555102	C10H16	136	1244	523.66	1.22264	319.2 ± 12.04 ^b^	397.72 ± 65.51 ^a^	321.15 ± 28.39 ^b^	162.8 ± 8.95 ^c^	ND
10	gamma-Terpinene	C99854	C10H16	136	1252.6	537.425	1.22338	798.36 ± 46.88 ^ab^	959.40 ± 174.82 ^a^	694.42 ± 62.09 ^b^	376.85 ± 103.37 ^c^	363.76 ± 1.88 ^c^
11	beta-Ocimene	C13877913	C10H16	136	1262.8	554.278	1.21669	350.21 ± 24.18 ^a^	309.36 ± 47.74 ^a^	353.76 ± 35.68 ^a^	ND	ND
12	alpha-Terpinolene	C586629	C10H16	136	1293.4	608.06	1.22347	380.5 ± 38.39 ^a^	378.35 ± 52.89 ^a^	396.63 ± 45.51 ^a^	ND	ND
13	(-)-alpha-Cubebene	C17699148	C15H24	204	1461.5	896.588	1.46078	ND	ND	298.65 ± 50.11	ND	ND
Total								6132.48 ± 185.43 ^b^	6867.13 ± 988.97 ^ab^	7792.87 ± 523.67 ^a^	2441.36 ± 521.83 ^c^	1783.23 ± 187.08 ^c^
Alcohols (14)												
14	Ethanol	C64175	C2H6O	46.1	948.7	203.28	1.13249	5668.79 ± 313.26 ^a^	3083.78 ± 122.53 ^b^	2644.8 ± 23.57 ^c^	2360.59 ± 79.90 ^d^	3070.48 ± 9.19 ^b^
15	1-Propanol	C71238	C3H8O	60.1	1049.4	275.561	1.11122	617.85 ± 27.31 ^c^	1700.86 ± 54.53 ^a^	991.70 ± 9.92 ^b^	218.92 ± 5.37 ^d^	179.65 ± 19.95 ^d^
16	1-Propanol, 2-Methyl	C78831	C4H10O	74.1	1101.8	325.971	1.17165	2134.68 ± 101.19 ^a^	1288.78 ± 65.36 ^b^	672.74 ± 39.15 ^c^	1228.18 ± 44.10 ^b^	1326.62 ± 10.74 ^b^
17	2-Pentanol	C6032297	C5H12O	88.1	1129.2	359.482	1.20601	293.60 ± 13.14 ^a^	289.91 ± 8.31 ^a^	125.61 ± 4.20 ^d^	163.09 ± 17.90 ^c^	213.80 ± 15.32 ^b^
18	Butanol	C71363	C4H10O	74.1	1150.3	387.669	1.18446	3254.19 ± 110.22 ^a^	1933.6 ± 92.69 ^b^	845.77 ± 24.74 ^d^	1614.24 ± 64.01 ^c^	1869.15 ± 128.08 ^b^
19	1-Penten-3-ol	C616251	C5H10O	86.1	1165.3	409.006	0.94525	1041.83 ± 37.81 ^b^	812.63 ± 34.60 ^c^	124.59 ± 9.17 ^d^	1094.73 ± 31.45 ^ab^	1161.08 ± 99.94 ^a^
20	1-Butanol-3-methyl	C123513	C5H12O	88.1	1214.8	479.41	1.24542	2453.2 ± 119.96 ^a^	2086.64 ± 86.35 ^b^	1386.09 ± 10.50 ^c^	1036.41 ± 43.15 ^e^	1164.03 ± 23.37 ^d^
21	1-Pentanol	C71410	C5H12O	88.1	1264.9	557.804	1.51407	3363.46 ± 65.20 ^b^	1770.25 ± 36.61 ^d^	588.86 ± 4.82 ^e^	2649.25 ± 89.61 ^c^	3585.66 ± 33.66 ^a^
22	(Z)-2-penten-1-ol	C1576950	C5H10O	86.1	1348.7	690.575	0.94872	886.82 ± 58.28 ^b^	318.98 ± 9.17 ^c^	244.7 ± 10.11 ^d^	903.56 ± 45.98 ^b^	971.03 ± 8.60 ^a^
23	(E)-3-hexen-1-ol	C928972	C6H12O	100	1364.2	715.571	1.22548	ND	229.38 ± 2.88	ND	ND	ND
24	1-Hexanol	C111273	C6H14O	102	1373.7	731.416	1.64339	396.1 ± 32.99 ^bc^	1925.51 ± 37.02 ^a^	189.19 ± 75.70 ^d^	334.86 ± 21.65 ^c^	470.66 ± 8.81 ^b^
25	1-Octen-3-ol	C3391864	C8H16O	128	1459	891.294	1.16671	210.29 ± 16.74 ^a^	160.78 ± 7.97 ^bc^	142.32 ± 1.29 ^cd^	171.74 ± 20.13 ^b^	135.11 ± 5.22 ^d^
26	(R/S)-linalool	C78706	C10H18O	154	1559	1124.59	1.22807	1144.23 ± 43.25 ^a^	235.39 ± 17.74 ^d^	471.70 ± 16.83 ^b^	294.67 ± 10.98 ^c^	124.72 ± 8.97 ^e^
27	1,2-Ethanediol	C107211	C2H6O2	62.1	1649.8	1388.84	1.09028	527.14 ± 15.43 ^a^	294.92 ± 15.92 ^c^	223.31 ± 9.10 ^d^	281.23 ± 25.59 ^c^	445.41 ± 8.35 ^b^
Total								21,992.18 ± 773.06 ^a^	16,131.41 ± 365.78 ^b^	8651.37 ± 109.37 ^e^	12,351.49 ± 329.00 ^d^	14,717.41 ± 238.50 ^c^
Aldehydes (14)												
28	Acetaldehyde	C75070	C2H4O	44.1	719.2	120.706	0.96037	2429.67 ± 377.35 ^a^	1080.18 ± 19.26 ^c^	921.57 ± 45.16 ^c^	1808.5 ± 88.66 ^b^	2562.61 ± 81.35 ^a^
29	Propanal	C123386	C3H6O	58.1	797.7	143.017	1.14524	2192.35 ± 100.49 ^a^	805.55 ± 36.31 ^d^	453.99 ± 7.44 ^e^	1687.48 ± 74.82 ^c^	1926.43 ± 41.07 ^b^
30	2-Methylpropanal	C78842	C4H8O	72.1	814.9	148.462	1.28214	903.71 ± 198.07 ^c^	941.63 ± 32.19 ^c^	793.46 ± 33.23 ^c^	1225.49 ± 36.03 ^b^	1763.85 ± 48.78 ^a^
31	3-Methyl butanal	C590863	C5H10O	86.1	934.3	194.839	1.40791	587.95 ± 56.65 ^b^	285.12 ± 25.80 ^c^	692.23 ± 28.33 ^a^	269.16 ± 3.74 ^c^	657.74 ± 17.25 ^a^
32	n-Pentanal	C110623	C5H10O	86.1	995.1	233.307	1.42282	4569.01 ± 110.56 ^b^	2180.4 ± 47.57 ^d^	1474.45 ± 35.29 ^e^	4328.56 ± 131.10 ^c^	6033.98 ± 68.33 ^a^
33	(E)-2-butenal	C123739	C4H6O	70.1	1060	284.673	1.03465	ND	ND	ND	224.74 ± 10.01	ND
34	1-Hexanal	C66251	C6H12O	100	1094.6	317.632	1.26463	1443.67 ± 33.79 ^c^	647.47 ± 78.43 ^d^	735.31 ± 69.96 ^d^	2428.67 ± 621.03 ^b^	4173.67 ± 346.42 ^a^
35	(Z)-2-pentenal	C1576869	C5H8O	84.1	1106.4	331.41	1.09079	332.16 ± 23.72 ^d^	512.90 ± 37.67 ^c^	113.51 ± 2.89 ^e^	2629.97 ± 81.48 ^a^	2301.34 ± 50.19 ^b^
36	Heptaldehyde	C111717	C7H14O	114	1188.8	443.09	1.69856	1455.10 ± 83.25 ^c^	618.20 ± 23.44 ^d^	653.07 ± 16.70 ^d^	2757.34 ± 43.89 ^b^	2905.68 ± 30.63 ^a^
37	(E)-2-hexenal	C6728263	C6H10O	98.1	1226.7	497.029	1.51855	1424.83 ± 104.14 ^b^	642.22 ± 50.01 ^c^	403.79 ± 13.82 ^d^	664.11 ± 19.94 ^c^	1573.94 ± 112.84 ^a^
38	1-Octanal	C124130	C8H16O	128	1308.8	630.048	1.4066	961.48 ± 9.19 ^c^	594.95 ± 21.18 ^d^	381.82 ± 12.04 ^e^	1267.93 ± 100.45 ^a^	1147.52 ± 34.98 ^b^
39	(E)-2-Heptenal	C18829555	C7H12O	112	1343.2	681.915	1.25984	236.77 ± 48.11 ^b^	ND	ND	185.58 ± 10.18 ^c^	288.03 ± 8.78 ^a^
40	1-Nonanal	C124196	C9H18O	142	1399.8	776.766	1.9458	1344.54 ± 78.22 ^b^	1353.77 ± 23.95 ^b^	859.62 ± 29.49 ^c^	1643.48 ± 69.11 ^a^	1308.08 ± 14.03 ^b^
41	Benzaldehyde	C100527	C7H6O	106	1501.3	983.51	1.1521	506.93 ± 98.08 ^c^	445.38 ± 23.71 ^c^	454.42 ± 7.01 ^c^	1543.92 ± 122.67 ^b^	1717.14 ± 31.75 ^a^
Total								18,388.17 ± 504.05 ^c^	10,107.77 ± 235.25 ^d^	7937.24 ± 83.09 ^e^	22,664.93 ± 1175.36 ^b^	28,360 ± 525.62 ^a^
Ketones (12)												
42	2-Propanone	C67641	C3H6O	58.1	827.7	152.6	1.11415	10,864.38 ± 428.90 ^a^	6316.76 ± 219.56 ^c^	5394.9 ± 63.27 ^d^	6470.36 ± 149.97 ^c^	8878.83 ± 164.14 ^b^
43	2-Butanone	C78933	C4H8O	72.1	926.8	190.557	1.24866	8996.62 ± 540.34 ^a^	5647.93 ± 200.03 ^d^	3691.97 ± 57.08 ^e^	6352.38 ± 198.47 ^c^	8241.88 ± 118.65 ^b^
44	2-Pentanone	C107879	C5H10O	86.1	993.9	232.443	1.37073	1687.87 ± 141.93 ^a^	364.47 ± 20.25 ^d^	367.47 ± 12.31 ^d^	574.76 ± 85.39 ^c^	1064.59 ± 28.96 ^b^
45	2-Hexanone	C591786	C6H12O	100	1094.4	317.389	1.19465	420.12 ± 10.89 ^a^	390.48 ± 2.98 ^b^	168.41 ± 10.66 ^d^	169.86 ± 14.86 ^d^	277.64 ± 14.10 ^c^
46	2-Heptanone	C110430	C7H14O	114	1185.5	438.739	1.63522	1427.63 ± 73.80 ^b^	2291.66 ± 86.90 ^a^	630.75 ± 18.77 ^d^	607.15 ± 49.85 ^d^	825.53 ± 37.13 ^c^
47	Cyclopentanone	C120923	C5H8O	84.1	1190.3	445.209	1.1031	715.79 ± 13.72 ^a^	ND	294.83 ± 7.44 ^b^	129.62 ± 8.42 ^d^	209.35 ± 4.17 ^c^
48	2-Butanone, 3-hydroxy	C513860	C4H8O2	88.1	1304.9	624.53	1.32957	4233.03 ± 105.96 ^a^	3268.00 ± 169.02 ^b^	2971.44 ± 41.25 ^c^	1462.07 ± 185.13 ^e^	2365.22 ± 10.61 ^d^
49	1-Hydroxy-2-propanone	C116096	C3H6O2	74.1	1322.1	649.609	1.23306	6678.39 ± 178.51 ^a^	4144.42 ± 189.44 ^c^	3869.84 ± 77.22 ^c^	4151.62 ± 259.60 ^c^	5368.88 ± 27.30 ^b^
50	3-Nonanone	C925780	C9H18O	142	1351.9	695.775	1.38179	227.86 ± 29.56 ^b^	ND	ND	ND	513.72 ± 10.86 ^a^
51	2-Methyl-2-hepten-6-one	C110930	C8H14O	126	1354.5	699.927	1.17987	465.14 ± 35.22 ^c^	486.34 ± 36.93 ^c^	2253.57 ± 71.97 ^a^	2348.93 ± 202.82 ^a^	938.37 ± 14.63 ^b^
52	Isomenthone	C491076	C10H18O	154	1481.6	939.477	1.33722	ND	ND	ND	151.56 ± 17.97	ND
53	1-(2-Furanyl)ethanone	C1192627	C6H6O2	110	1497.3	974.469	1.11292	ND	191.46 ± 14.10 ^a^	102.35 ± 2.76 ^b^	ND	ND
Total								35,716.85 ± 1429.39 ^a^	23,101.51 ± 630.4 ^c^	19,745.55 ± 207.58 ^d^	22,418.32 ± 788.07 ^c^	28,684.02 ± 296.18 ^b^
Esters (7)												
54	Ethyl formate	C109944	C3H6O2	74.1	811.7	147.429	1.21721	1380.42 ± 273.22 ^a^	822.53 ± 31.59 ^b^	485.65 ± 18.90 ^c^	1361.17 ± 59.04 ^a^	1575.19 ± 105.53 ^a^
55	Acetic acid ethyl ester	C141786	C4H8O2	88.1	899.8	178.347	1.33899	6019.39 ± 492.26 ^a^	5438.97 ± 195.53 ^b^	4850.66 ± 73.53 ^c^	2534.74 ± 334.47 ^e^	4091.99 ± 105.43 ^d^
56	2-Methyl-1-propyl acetate	C110190	C6H12O2	116	1025.2	255.893	1.62184	2538.14 ± 180.90	ND	ND	ND	ND
57	Acetic acid butyl ester	C123864	C6H12O2	116	1082.7	305.156	1.62919	ND	3516.67 ± 118.09 ^a^	3882.44 ± 104.33 ^a^	ND	ND
58	Acetic acid hexyl ester	C142927	C8H16O2	144	1288	598.162	1.38879	ND	267.68 ± 11.73	ND	ND	ND
59	Ethyl 2-hydroxypropanoate	C97643	C5H10O3	118	1365.6	717.885	1.14167	ND	ND	758.95 ± 25.54	ND	ND
60	Bornyl acetate	C76493	C12H20O2	196	1589.1	1206.11	1.22531	301.52 ± 21.14 ^a^	105.78 ± 6.77 ^c^	84.66 ± 2.10 ^c^	192.73 ± 18.35 ^b^	ND
Total								10,239.47 ± 486.72 ^a^	10,151.62 ± 260.86 ^a^	10,062.36 ± 149.75 ^a^	4088.64 ± 396.87 ^c^	5667.18 ± 159.14 ^b^
Acids (2)												
61	Acetic acid	C64197	C2H4O2	60.1	1457.3	887.858	1.05071	5664.27 ± 408.35 ^a^	2726 ± 180.60 ^c^	2074.82 ± 87.14 ^d^	2681.67 ± 141.01 ^c^	4204.67 ± 198.31 ^b^
62	Propanoic acid	C79094	C3H6O2	74.1	1543.3	1084.29	1.10895	835.84 ± 79.33 ^a^	412.78 ± 11.75 ^c^	293.98 ± 19.10 ^d^	340.36 ± 17.51 ^cd^	659.19 ± 49.19 ^b^
Total								6500.11 ± 486.77 ^a^	3138.78 ± 191.34 ^c^	2368.81 ± 104.77 ^d^	3022.03 ± 149.28 ^c^	4863.85 ± 247.46 ^b^
S-containings (5)												
63	Dimethyl sulfide	C75183	C2H6S	62.1	787.3	139.857	0.96225	2346.34 ± 106.48 ^a^	802.86 ± 74.33 ^c^	859.51 ± 26.64 ^c^	782.33 ± 44.55 ^c^	1082.38 ± 22.67 ^b^
64	Allyl sulfide	C592881	C6H10S	114	1154.6	393.751	1.12953	167.36 ± 12.33 ^a^	122.31 ± 5.44 ^b^	71.59 ± 4.95 ^c^	ND	ND
65	Methyl propyl disulfide	C2179604	C4H10S2	122	1238.4	514.905	1.14557	ND	491.72 ± 40.24 ^a^	153.5 ± 7.48 ^b^	ND	ND
66	2-Methyl-3-furanthiol	C28588741	C5H6OS	114	1334.3	668.209	1.14584	ND	ND	152.57 ± 2.05	ND	ND
67	3-(Methylsulfanyl)propanal	C3268493	C4H8OS	104	1452.3	877.523	1.09232	ND	152.63 ± 24.34 ^c^	ND	293.37 ± 32.42 ^b^	305.86 ± 6.78 ^a^
Total								2513.70 ± 111.91 ^a^	1569.51 ± 15.04 ^b^	1237.16 ± 35.84 ^d^	1075.71 ± 61.1 ^e^	1388.25 ± 16.00 ^c^
N-containings (3)												
68	Pyrazine	C290379	C4H4N2	80.1	1221.4	489.083	1.05134	900.48 ± 14.45 ^a^	666.45 ± 25.12 ^b^	463.29 ± 6.19 ^c^	181.88 ± 16.87 ^d^	194.5 ± 6.42 ^d^
69	2,3-Dimethylpyrazine	C5910894	C6H8N2	108	1364.2	715.61	1.1172	ND	203.19 ± 28.76 ^b^	424.44 ± 33.09 ^a^	ND	ND
70	2-Ethyl-6-methylpyrazine	C13925036	C7H10N2	122	1398.1	773.708	1.17689	ND	ND	267.90 ± 6.3	ND	ND
Total								900.48 ± 14.45 ^b^	869.64 ± 53.71 ^b^	1155.63 ± 36.09 ^a^	181.88 ± 16.87 ^c^	194.50 ± 6.42 ^c^
Others (3)												
71	1,8-Cineole	C470826	C10H18O	154	1201.5	460.497	1.29465	5976.96 ± 333.04 ^b^	3626.33 ± 55.43 ^c^	3237.58 ± 48.67 ^d^	5700.71 ± 209.76 ^b^	6786.82 ± 79.04 ^a^
72	2-Pentyl furan	C3777693	C9H14O	138	1243.2	522.409	1.25713	274.97 ± 10.01 ^b^	425.40 ± 106.26 ^a^	146.18 ± 18.58 ^c^	179.50 ± 60.18 ^bc^	273.20 ± 13.00 ^b^
73	2-Methyl-3-ketotetrahydrofuran	C3188009	C5H8O2	100	1283.3	589.748	1.43062	1850.32 ± 84.69 ^a^	698.99 ± 14.36 ^c^	1507.03 ± 7.41 ^b^	586.80 ± 11.53 ^d^	730.50 ± 8.63 ^c^
Total								8102.26 ± 406.49 ^a^	4750.72 ± 92.58 ^c^	4890.78 ± 72.73 ^c^	6467.01 ± 267.90 ^b^	7790.52 ± 99.41 ^a^

Note: Data are presented as mean ± standard deviation (*n* = 3). Mean values with different letters (^a–e^) indicate significant differences (*p* < 0.05).

**Table 6:** Potentially relevant aroma-active compounds (*OAV* ≥ 1 and VIP > 1) identified in five sauced beef samples.

No.	Compound	Category	Odor Threshold (µg/kg) ^A^	*OAV*				
				B1	B2	B3	B4	B5
1	1-Hexanol	Alcohol	34	11.65	56.63	5.56	9.85	13.84
2	1-Octen-3-ol	Alcohol	1	210.29	160.78	142.32	171.74	135.11
3	(R/S)-Linalool	Alcohol	2.4	476.76	98.08	196.54	122.78	51.97
4	(E)-3-Hexen-1-ol	Alcohol	110	—	2.09	—	—	—
5	2-Methyl-3-furanthiol	S-containing	0.001	—	—	152,570.00	—	—
6	2-Methylpropanal	Aldehyde	1	903.71	941.63	793.46	1225.49	1763.85
7	3-Methyl butanal	Aldehyde	0.35	1679.86	814.63	1977.80	769.03	1879.26
8	(E)-2-Hexenal	Aldehyde	3.1	459.62	207.17	130.25	214.23	507.72
9	1-Nonanal	Aldehyde	3.1	433.72	436.70	277.30	530.15	421.96
10	2-Heptanone	Ketone	3.5	407.89	654.76	180.21	173.47	235.87
11	2-Methyl-2-hepten-6-one	Ketone	6.3	73.83	77.20	357.71	372.85	148.95
12	Isomenthone	Ketone	40.451	—	—	—	3.75	—
13	Acetic acid hexyl ester	Ester	17	—	15.75	—	—	—
14	2-Pentylfuran	Other	270	1.02	1.58	—	—	1.01
Total potential aroma-active compounds number				10	12	10	10	10

Note: — = not identified as potential aroma-active compound. “^A^” = the odor threshold of the compound is sourced from the website https://www.doc88.com/p-2039102934971.html (accessed on 2 September 2025).

**Table 7:** Texture profile analysis of five sauced beef samples.

Sample Number	Hardness (g)	Cohesiveness	Springiness (mm)	Gumminess (g)	Chewiness (g)
B1	2541.25 ± 182.82 ^a^	0.40 ± 0.02 ^bc^	3.20 ± 0.28 ^a^	1279.63 ± 43.68 ^a^	4079.50 ± 240.70 ^a^
B2	1436.63 ± 140.22 ^b^	0.58 ± 0.04 ^a^	2.93 ± 0.09 ^ab^	972.88 ± 15.70 ^b^	2463.25 ± 140.97 ^b^
B3	1263.44 ± 132.61 ^b^	0.28 ± 0.00 ^d^	2.70 ± 0.22 ^bc^	470.38 ± 41.26 ^d^	1317.63 ± 124.04 ^d^
B4	1150.94 ± 30.84 ^c^	0.41 ± 0.01 ^b^	2.95 ± 0.11 ^ab^	541.50 ± 13.08 ^c^	1651.88 ± 116.17 ^c^
B5	1138.50 ± 93.44 ^c^	0.37 ± 0.01 ^c^	2.58 ± 0.04 ^c^	480.25 ± 9.03 ^d^	1262.00 ± 120.64 ^d^

Note: Values are expressed as mean ± standard deviation. Within each column, different lowercase letters indicate significant differences (*p* < 0.05).

**Table 8:** Statistics of sensory scores of evaluation indicators of different sauced beef samples.

Sample Number	Color			Taste			Flavor			Texture			Acceptability		
	*V*_1_	*V*_2_	*V*_3_	*V*_1_	*V*_2_	*V*_3_	*V*_1_	*V*_2_	*V*_3_	*V*_1_	*V*_2_	*V*_3_	*V*_1_	*V*_2_	*V*_3_
B1	1	9	0	2	8	0	4	6	0	5	5	0	5	5	0
B2	8	2	0	6	4	0	6	4	0	7	3	0	5	5	0
B3	8	2	0	4	6	0	3	7	0	7	3	0	5	5	0
B4	3	7	0	9	1	0	10	0	0	1	9	0	5	5	0
B5	6	4	0	4	6	0	8	2	0	5	5	0	4	6	0

Note: *V*_1_ = excellent (90–100); *V*_2_ = good (80–89); and *V*_3_ = poor (70–79).

## Data Availability

The original contributions presented in this study are included in the article/Supplementary Material. Further inquiries can be directed to the corresponding authors.

## Supplementary Materials

The following supporting information can be downloaded at: https://www.mdpi.com/article/10.3390/foods15213961/s1, Table S1. Sensitivity analysis of FCE scores and sample rankings under different weighting schemes.

## Abbreviations

The following abbreviations are used in this manuscript:

E-nose	Electronic nose
GC-IMS	gas chromatography–ion mobility spectrometry
FCE	Fuzzy comprehensive evaluation
VOCs	Volatile organic compounds
PCA	Principal component analysis
PLS-DA	Partial least squares discriminant analysis
TPA	Texture profile analysis
VIP	Variable importance in projection
OAV	Odor activity value
LAV	Laboratory Analytical Viewer
ANOVA	Analysis of variance
