# Supplementary material for: Comparative Analysis of Sauced Beef Based on Electronic Nose, GC-IMS, and Fuzzy Comprehensive Evaluation
Source: Foods. 2026 Nov 4;15(21):3961. doi: 10.3390/foods15213961 (PMC13762807; doi:10.3390/foods15213961)
Supplement: Supplementary file 1 [file foods-15-03961-s001.zip › foods-4614566-supplementary.pdf]

**Table S1.** Sensitivity analysis of FCE scores and sample rankings under different weighting schemes.

| Weighting Scenario | Color | Taste | Flavor | Texture | Acceptability | B1 | B2 | B3 | B4 | B5 | Sample Ranking |
|---|---|---|---|---|---|---|---|---|---|---|---|
| Equal weights | 0.20 | 0.20 | 0.20 | 0.20 | 0.20 | 83.00 | 86.40 | 85.40 | 85.60 | 85.40 | B2>B4>B3=B5>B1 |
| Flavor-prioritized | 0.15 | 0.25 | 0.30 | 0.15 | 0.15 | 83.05 | 86.30 | 84.90 | 86.60 | 85.65 | B4>B2>B5>B3>B1 |
| Texture-prioritized | 0.15 | 0.20 | 0.20 | 0.30 | 0.15 | 83.00 | 86.45 | 85.45 | 85.30 | 85.40 | B2>B3>B5>B4>B1 |
| Excluding acceptability | 0.25 | 0.25 | 0.25 | 0.25 | 0.00 | 82.50 | 86.75 | 85.50 | 85.75 | 85.75 | B2>B4=B5>B3>B1 |

Note: The comprehensive scores were calculated using ($Y$=$X$\times $R$) and ($T$=$Y$\times $K$), where ($X$) represents the attribute weighting vector, ($R$) represents the fuzzy evaluation matrix, ($Y$) represents the overall sensory evaluation vector, and ($K$=(90,80,70)^T) represents the evaluation scores corresponding to the "excellent," "good," and "poor" categories, respectively. Bold values indicate the highest comprehensive score under each weighting scenario.
